# Supernumerary Digits of the Hand

**Published:** 2016-01-15

**Authors:** Daniel Delgadillo, Nicholas S. Adams, John A. Girotto

**Affiliations:** ^a^Michigan State University College of Human Medicine, Grand Rapids, Mich; ^b^Plastic and Reconstructive Surgery Residency, Grand Rapids Medical Education Partners, Grand Rapids, Mich; ^c^Pediatric Plastic and Craniofacial Surgery, Helen DeVos Children's Hospital, Grand Rapids, Mich

**Keywords:** polydactyly, duplication, supernumerary digits, hand embryology, congenital hand anomaly

## DESCRIPTION

**Figure 1:**
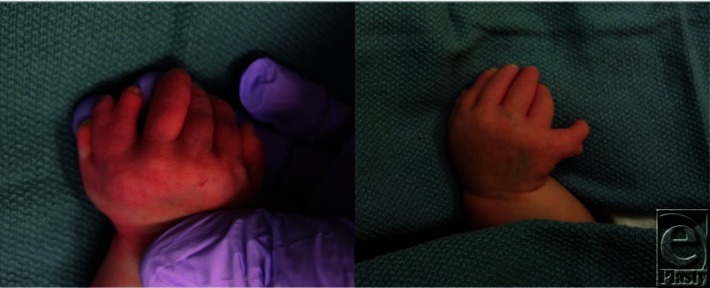
Left and right hands with ulnar polysyndactyly. Categorized as type V under the Duran classification.

**Figure 2:**
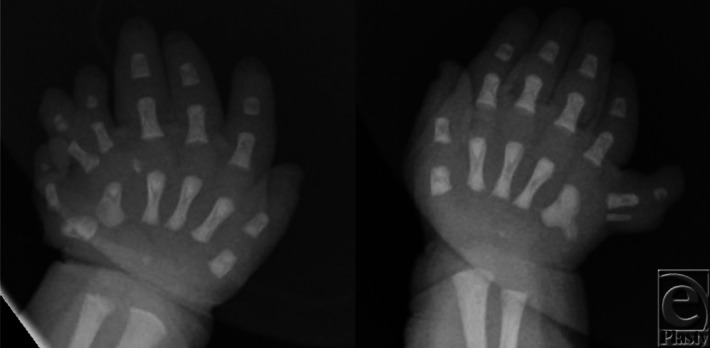
Anteroposterior x-ray images of the same patient.

A 3-day-old female patient was evaluated in the neonatal intensive care unit for ‘extra fingers’ on bilateral upper extremities (Fig 1). X-ray images were taken (Fig 2) and genetic testing was done on the basis of numerous congenital abnormalities. The patient received a diagnosis of Ellis-van Creveld syndrome.

## QUESTIONS

1. **How is polydactyly defined?**
2. **What syndromes and patient populations are associated with polydactyly?**
3. **What are the major classifications of polydactyly?**
4. **How does the embryonic limb develop?**

## DISCUSSION

The most common congenital anomaly of the hand is polydactyly, characterized by partial or complete duplication of a digit on a normal 1-thumb and 4-finger hand.^1^ These supernumerary digit anomalies can be classified into 3 distinct categories on the basis of their location: postaxial (ulnar), preaxial (radial), and central polydactyly. Each subset of polydactyly has its own specific genetic and ethnic association, with some being more heterogeneous than others.

Certain ethnicities and races have higher prevalence of polydactyly. African Americans have a 10-fold risk of ulnar polydactyly with an incidence of 1 in 143. Conversely, radial duplication is more common in whites but overall less common.^2^ Currently, more than 300 polydactyly disorders have been identified, with a majority being of syndromic association. These encompass all patterns of Mendelian inheritance as well as non-Mendelian forms. Certain syndromes are associated with specific forms of polydactyly. For example, trisomy 13 and Ellis-van Creveld and Smith-Lemli-Opitz syndromes are highly associated with ulnar polydactyly. Alternatively, Fanconi pancytopenia and Townes-Brocks and Holt-Oram syndromes are most frequently seen with radial anomalies.^3^

Postaxial polydactyly is the most common of these categories, with the presence of supernumerary digit(s) on the ulnar side of the hand. Many classification systems exist for describing postaxial polydactyl. A new system recently proposed by Duran et al^4^ subclassifies the digital anomalies based on anatomical features of the duplication. Type I is characterized by a skin nub that contains no nail or bone, whereas type II is defined by a hypoplastic proximal phalanx. Type III involves malformation of the proximal phalanx and is further divided into IIIA representing a bifid proximal phalanx and IIIB a duplicated proximal phalanx. Type IV consists of metacarpal malformations and can be further divided as follows: IV-A, fusion of the metacarpal; IV-B, bifid metacarpal; and IV-C, duplication of the metacarpal. Type V (complicated type) includes triplication of the small finger, polysyndactyly, or both.^4^ The second most common congenital duplication is preaxial, or radial polydactyly. The incidence of radial polydactyly is estimated to be 1 in 3000 live births.^1^^,^^2^ The most widely accepted and utilized classification system for preaxial polydactyly is the Wassel classification system, which is divided into 7 classes progressing distal to proximal. The even types (2, 4, and 6) bifurcate at the joints (distal interphalangeal, metacarpophalangeal, and carpometacarpal, respectively), with type 4 being most common at 40%.^5^ Central polydactyly is the least common and is the presence of a supernumerary digit between the thumb and the fifth digit, most commonly at the ring finger. It is most often found in conjunction with syndactyly.^1^^,^^2^^,^^6^ Previous studies of central polydactyly cases suggest an autosomal dominant pattern of inheritance.^5^

**Figure 3:**
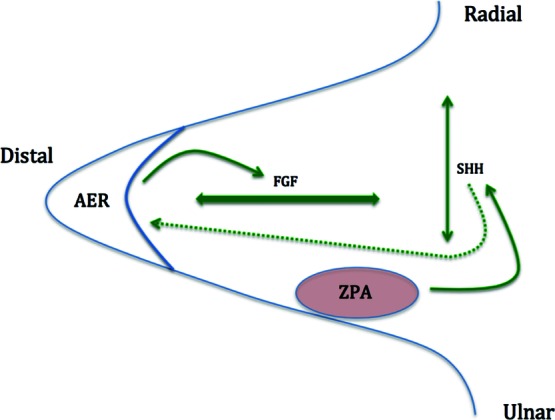
Proximal-distal and radioulnar axis development of the limb bud occurring between gestational weeks 4 and 8. AER releases FGF, allowing for growth along the proximal-distal axis. ZPA located on the lateral plate releases SHH, causing the formation of an ‘ulnarizing’ gradient and the radioulnar axis. SHH also induces AER to release FGF, indicated by the dotted green arrow. AER indicates apical ectodermal ridge; FGF, fibroblast growth factor; ZPA, zone of polarizing activity; and SHH, sonic hedgehog.

**Figure 4:**
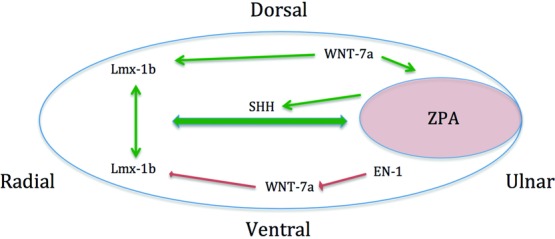
Cross-sectional view of the limb bud during weeks 4 to 8 of gestation, highlighting maturation of the dorsal-ventral axis. Dorsal ectoderm Wingless-type (WNT) signaling center expresses WNT-7a, which activates ZPA and induces expression of LIM Homeobox (Lmx-1b). Lmx-1b expression drives dorsoventral growth indicated by the green arrows. However, engrailed-1 (EN-1) expression blocks WNT-7a, leading to decreased Lmx-1b (red arrows) on the volar side allowing for surface differentiation. ZPA indicates zone of polarizing activity.

Embryological development of the upper limbs begins shortly after week 4 of gestation. It is at this time that the limb is most susceptible to congenital abnormalities caused by disruption of the signaling centers and/or aberrant production of key regulatory proteins. Three critical signaling centers have been identified to play a substantial role in embryological development. These lead to the asymmetric development of the hand and include the apical ectodermal ridge (AER), zone of polarizing activity (ZPA), and the dorsal ectoderm Wingless-type signaling center (WNT).^1^^,^^2^ AER is a highly specialized ectodermal region that expresses critical fibroblast growth factor family (FGF-2,-4,-8) molecules, which contribute to the growth along the proximal-distal axis and interdigital apoptosis.^1^^,^^2^ Formation and growth along the anterior-to-posterior (radioulnar) axis is tightly regulated by sonic hedgehog (SHH). SHH forms a gradient through diffusion originating from the ZPA, which is located within the lateral plate mesoderm in what will become the ulnar limb (Fig 3). Secretion of SHH also induces growth via AER by increasing expression of FGF-4.^7^ Dorsoventral differentiation (Fig 4) is controlled by WNT-7a protein expression from the WNT signaling center in the dorsal ectoderm. WNT-7a induces LIM Homeobox (Lmx-1b) gene expression resulting in dorsoventral limb growth.^6^ Complementary protein Engrailed-1 blocks WNT-7a and allows for specific ventral (volar) surface differentiation.^1^^,^^2^ Despite advancement of gene identification and interaction in limb development, the molecular mechanism of polydactyly remains unclear but is likely heterogeneous.

## References

[1] Guo B, Lee SK, Paksima N (2013). Polydactyly: a review. Bull Hosp Jt Dis (2013).

[2] Watt AJ, Chung KC (2009). Duplication. Hand Clin.

[3] Biesecker LG (2011). Polydactyly: how many disorders and how many genes? 2010 update. Dev Dyn.

[4] Duran A, Ciloglu NS, Buyukdogan H (2015). A classification system for ulnar polydactyly and clinical series. J Hand Surg.

[5] Choi M, Sharma S, Louie O, Thorne C (2007). Congenital hand abnormalities. Grabb and Smith's Plastic Surgery.

[6] Riddle RD, Ensini M, Nelson C, Tsuchida T, Jessell TM, Tabin C (1995). Induction of the LIM homeobox gene Lmx1 by WNT6a establishes dorsoventral pattern in the vertebrate limb. Cell.

[7] Laufer E, Nelson CE, Johnson RL, Morgan BA, Tabin C (1994). Sonic hedgehog and fgf-4 act through a signaling cascade and feedback loop to integrate growth and patterning of the developing limb bud. Cell.

